# Identifying spatiotemporal patterns of COVID-19 transmissions and the drivers of the patterns in Toronto: a Bayesian hierarchical spatiotemporal modelling

**DOI:** 10.1038/s41598-022-13403-x

**Published:** 2022-06-07

**Authors:** Nushrat Nazia, Jane Law, Zahid Ahmad Butt

**Affiliations:** 1 School of Public Health Sciences, University of Waterloo, 200 University Ave., Waterloo, ON N2L3G1 Canada; 2 School of Planning, University of Waterloo, 200 University Ave., Waterloo, ON N2L3G1 Canada

**Keywords:** Environmental sciences, Diseases, Risk factors

## Abstract

Spatiotemporal patterns and trends of COVID-19 at a local spatial scale using Bayesian approaches are hardly observed in literature. Also, studies rarely use satellite-derived long time-series data on the environment to predict COVID-19 risk at a spatial scale. In this study, we modelled the COVID-19 pandemic risk using a Bayesian hierarchical spatiotemporal model that incorporates satellite-derived remote sensing data on land surface temperature (LST) from January 2020 to October 2021 (89 weeks) and several socioeconomic covariates of the 140 neighbourhoods in Toronto. The spatial patterns of risk were heterogeneous in space with multiple high-risk neighbourhoods in Western and Southern Toronto. Higher risk was observed during Spring 2021. The spatiotemporal risk patterns identified 60% of neighbourhoods had a stable, 37% had an increasing, and 2% had a decreasing trend over the study period. LST was positively, and higher education was negatively associated with the COVID-19 incidence. We believe the use of Bayesian spatial modelling and the remote sensing technologies in this study provided a strong versatility and strengthened our analysis in identifying the spatial risk of COVID-19. The findings would help in prevention planning, and the framework of this study may be replicated in other highly transmissible infectious diseases.

## Introduction

COVID-19, caused by the coronavirus SARS-CoV-2, has complex transmission dynamics possibly generated by different risk factors such as demographic, social and environmental factors^1–4^. It is highly transmissible by either direct contact with an infected individual or transmission via contaminated surfaces leaving the world in the last two years at a halt in many aspects^5^. In Canada, over 3.4 million COVID-19 cases and over 37,485 COVID-19 related deaths have been reported, with Ontario and Quebec reporting the highest cumulative cases in the nation^6^. Over 11.2 billion COVID-19 vaccine doses have been administered around the world^7^, and at least 89% population over the age of 5 years in Canada have received at least one dose (March 28, 2022)^8^. In Canada, Toronto continues to experience substantial COVID-19 incidence and hospitalization rates despite several interventions and mitigation efforts made by the local and provincial public health officials^9,10^.

As momentum grows to end this global pandemic, understanding the disease trends, detecting hotspots, and identifying important risk factors at the community level is an imperative research effort. Temperature is often a significant risk factor for infectious diseases because a certain temperature can help a virus evolve rapidly^11^. A county-level study in the USA found a strong negative influence of nighttime land surface temperature (LST) with COVID-19 using low-resolution Moderate Resolution Imaging Spectroradiometer (MODIS) images of 2020^12^. Another study found LST to be an important determining factor in the COVID-19 infection rate in Kolkata, India^13^. Another study by Hassan et al. identified a strong positive relationship between COVID-19 and LST^14^. This study showed that a 1 °C increase in LST is linked with a 36.1% increase in COVID-19 incidence rates in Bangladesh. Many prior studies have discussed the associations between temperature and COVID-19^15^; however, most of these studies have used short temporal periods, lower resolution images or have not performed a small area analysis. The results were diverse and often contradictory in different geographic areas, leaving a gap in understanding the impact of temperature on COVID-19 transmission in a small urban area.

Previous studies have also linked different socioeconomic and demographic factors to explain the heterogeneity in COVID-19 rates across space. Some studies have found that areas with low socioeconomic status, such as rate of poverty^16–20^, rate of education^16,19,21,22^, ethnicity status^16,20,23–28^, immigration status^29–33^, unemployment rate^21,27,34^, and housing conditions^21,22,30^, tend to experience higher rates of COVID-19 infections and morbidity due to the economic and health inequalities. A previous work by Vaz in 2021^35^ found that the COVID-19 cases are not uniformly distributed in Toronto. Social injustice, socioeconomic vulnerability and population density were found to be related to the increasing spread and incidence of COVID-19. A work by Feng in 2021 has found that neighbourhood-level population density and low income have a significant effect on COVID-19 mortality risk^36^. Another study of Toronto neighbourhoods by Choi et al. in 2021 found that several demographic and socioeconomic factors such as higher education rate, lower rates of immigrants (foreign-born residents) were significantly associated with decreasing the number of COVID-19 infections^37^. These past studies implied that these factors might disproportionately impact COVID-19 infection rates. Finally, analyzing the spatiotemporal trends of COVID-19 transmission to understand whether the disease risk trends show increasing, decreasing or stable patterns over the study period has also been understudied.

In this study, we used a Bayesian hierarchical spatiotemporal models to investigate the spatiotemporal patterns of COVID-19 transmission in Toronto. The approach allows us to deal with uncertainties related to the data, the process and model parameters^38^ since it has the capacity to account for missing data, measurement errors and ecological bias^39,40^. Even though Bayesian models have a clear advantage, only a handful of studies^41–45^ in COVID-19 research has adopted Bayesian approaches to predict COVID-19 risk, identify trends and locate hotspots. Motivated by the recent increase in COVID-19 incidence in winter, this study scrutinizes the effect of weather and socioeconomic and demographic factors on COVID-19 using a small area analysis. Within the scope of this study, we will answer four research questions: (1) where were the hotspots of COVID-19, (2) what was the temporal patterns of risk in the study area, (3) was there a relationship between land surface temperature and COVID-19 while adjusting for socioeconomic and demographic factors, and (4) was there any spatiotemporal trend of COVID-19 transmission in Toronto (e.g., stable, increasing or decreasing)?

## Methods

### The study area

**Figure 1:**
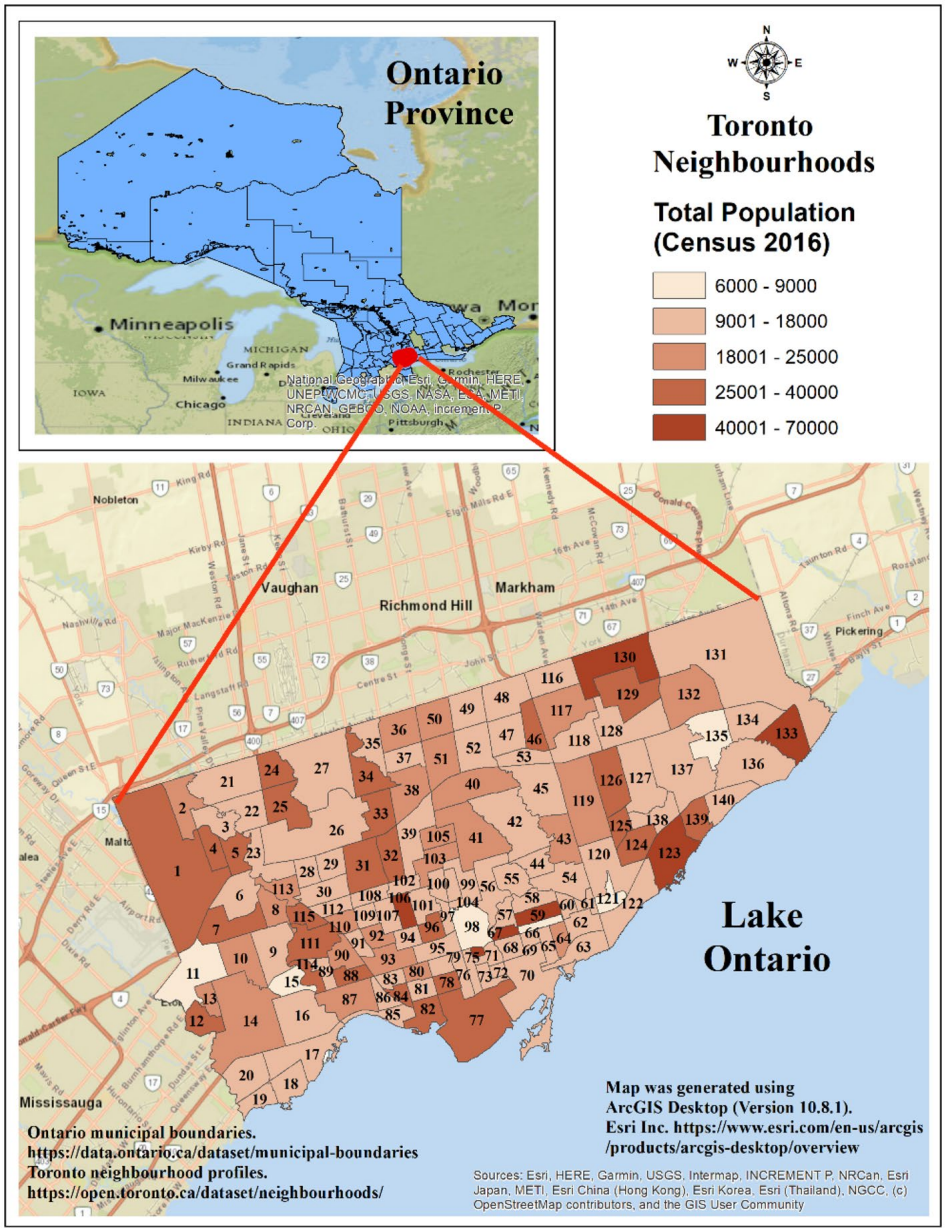
Study area in Toronto, Ontario, Canada. The numbers inside the neighbourhoods represent the neighbourhood ID.

The study area is the metropolitan city of Toronto, located on the northwestern shore of Lake Ontario at an altitude of 175 m (43° 42′ 00″ N latitude and 79° 24′ 58″ W longitude). It is the capital city of the province of Ontario in Canada, with a total land area of 630 km^2^ and a high population density of 4692 persons/km^21^. Toronto has a well-defined urban heat island with warmer temperatures, mostly at night and in winter, compared to the rest of the city’s surrounding regions^2–4^. The average temperature of Toronto is 21.9 °C (81.3 *°*F), and the annual rainfall is 845 mm (33.3 in.), with July (17–25 °C/62 °F to 77 °F) the hottest and February (average − 4.4 °C/24.1 °F) the coldest months of the year^5^. The map of the study area was created using ArcGIS Desktop software^46^ version 10.8.1 is provided in Fig. 1.

### Geographic boundary, population, and case data

There were 140 geographically distinct neighbourhoods in the study area. The digital data of the geographic boundaries for these neighbourhoods were acquired from the open data portal of the city of Toronto^47^. We used the COVID-19 case data originally collected by Toronto Public Health and extracted from the provincial Case & Contact Management System (CCM)^6^ by the city of Toronto. The case dataset contains the demographic, geographic, and severity information for all confirmed and probable, sporadic, and outbreak-associated cases. We aggregated the daily case data from January 2020 to October 2, 2020, by the 89 epidemiological weeks and at the neighbourhood level. The total population for each neighbourhood was collected from neighbourhood profiles using the census 2016 population dataset collected and released by Statistics Canada^48^.

### Demographic and socioeconomic variables

Previous studies have also linked different socioeconomic and demographic factors to explain the heterogeneity in COVID-19 rates across space^13,14,16–29^. Based on these past studies and data availability, we selected 11 potential demographic and socioeconomic variables under six categories (demographic, core housing need, race/ethnicity/minority status, education, economic and immigration status) for our model. These data were collected from the Toronto neighbourhood profile based on the 2016 census from Statistics Canada^8^. The full descriptions of the variables are provided in Table 1.

**Table 1:** Descriptions of the demographic and socioeconomic covariates (2016 Census).

Category	Covariate	Covariate description
Education	Higher education rate	Percentage of the population who are aged 25–64 years and have a higher level of education (having a university certificate, diploma or at least a bachelor's degree)^49^
Economic	Unemployment rate	Percentage of population in private households who are over 15 years and not employed in the labour force)^50^
	Prevalence of low-income	The percentage of the population whose income falls below the low-income cut-off (LICO) table represents the poverty line^51,52^ The cut-off thresholds represent the income levels at which these families or persons were expected to spend 20 percentage points or more of their after-tax income than average on food, shelter and clothing^51^
Core housing need	Rate of unaffordable housing	Percentage of households in a neighbourhood costs ≥ 30% of total before-tax income ton adequate shelter^53^
	Rate of inadequate housing	Percentage of population in the neighbourhood not requiring any major repairs as reported by the residents^53^
	Rate of unsuitable housing	Percentage of population in the neighbourhood without suitable accommodations according to the National Occupancy Standard (NOS) standards^53^
Race/ethnicity/minority status	Percentage of black population	The percentage of black population in a neighbourhood
	Visible minority	Percentage of visible minority in a neighbourhood. Visible minority refers to a person who belongs to a visible minority group as defined by the Employment Equity Act, defining visible minorities as "persons, other than Aboriginal peoples, who are non-Caucasian in race or non-white in colour”^54^
	Immigrants	Percentage of the population born outside of Canada and who is, or who has ever been, a landed immigrant or a permanent resident^55^
Demographic	Population density	The number of persons per square kilometre in a neighbourhood

### The land surface temperature (LST) data

We used Landsat imageries to retrieve the weekly average land surface temperature (LST) at the neighbourhood level for 89 consecutive weeks (January 19, 2021–October 2, 2022). The timing of these imageries was approximately 4 PM (GMT). We collected mostly Landsat 8 imageries (75), and if the imageries for a particular week were not available in the Landsat 8, we used Landsat 7 imageries instead. However, no images were available for two epidemiological weeks (week 45 of 2000 and week 7 of 2021). We used the average temperature of the previous and following week for these two weeks. The images were corrected using atmospheric correction parameters collected by the National Aeronautics and Space Administration (NASA) to improve estimation accuracy. The complete details of the LST retrieval process, image data, acquisition time, atmospheric parameters are summarised in Table S1 in Appendix 1 (Supplementary Information 2).

### Zonal statistics

Zonal statistics was performed in ArcGIS Desktop software^46^ version 10.8.1 to calculate the average LST values for Retrieval of spectral radiance. We applied a mask comprised of the polygons (neighbourhood boundaries) from the map of the city of Toronto and used the zonal statistics to calculate the average LST of all pixels by neighbourhood.

### Variable selection

Variable selection was conducted using a two-step method to fit into the multivariable regression model. In the first step, we performed a Pearson’s correlation and generated a correlation matrix taking account of all the potential risk factors to evaluate collinearity among these risk factors (Appendix 2, Fig. S1 in Supplementary Information 2). Note that the presence of collinearity among the independent variables can result in model overfitting, unstable estimates and inaccurate variances, and consequently incorrect inferences about associations between explanatory and the response variables^56–59^. We observed that the percentage of immigrants had a high correlation (> 0.7) with the percentage of the black population and the percentage of visible minorities. Since Choi et al.^37^, in a study conducted in our study area in Toronto, stated that the percentage of immigrants is an important risk factor in our study area in Toronto, we selected this variable over the percentage of the black population and percentage of visible minorities. The prevalence of low income was found to be strongly correlated with the rate of unaffordable housing and unemployment rate. Out of the three core housing need variables, the rate of unsuitable and the rate of unaffordable housing had a strong correlation with multiple variables. Since the rate of inadequate housing did not have a strong correlation with any other variables, we selected this (inadequate housing) variable over other housing variables. Among the correlated factors, we selected the prevalence of low income over the unemployment rate based on an earlier study in our study area that found low income to be strongly associated with COVID-19^36^. Finally since the LST and population density did not have a strong correlation with other factors, we kept both in the model.

In the second step, the six selected variables from the first step: land surface temperature (LST), the prevalence of low income, rate of higher education, inadequate housing, percentage of immigrants and population density per square kilometres, were fitted in a Bayesian variable selection method using BayesVarSel package^28^ in RStudio^60^ version 2021.09.0 to select the variables that fit best in our Bayesian hierarchical spatial model (Appendix 2, Table S2 in Supplementary Information 2). The approach uses priors as proposed by Bayarri et al.^61^, computes posterior probabilities of hypotheses or the models, and delivers tools in a coherent and complete analysis to properly summarise the outputs^62^. This approach yielded LST, higher education rate and immigrant variables with higher posterior probabilities and marginal inclusion probabilities (Appendix 2, Table S2), suggesting that these three variables are very relevant, highly influential, and the best fit for our Bayesian regression models.

### Standardization of the variables

Since the variables were in different units, such as raw values, percentages, and prevalence rates, we used the Z-transformation technique, where the mean for all values was subtracted from each value and was then divided by the standard deviation of the values of the variables to obtain standardized values for the Bayesian model.

### Bayesian spatiotemporal models

We performed four Bayesian hierarchical space–time models to investigate the long-term spatiotemporal effects of COVID-19 using two frameworks: space–time separable^38^ and the space–time inseparable models^38,63^. Model 1 drew the space–time separable framework, while Models 2, 3 and 4 drew the space–time inseparable modelling frameworks.

To model each outcome value ${y}_{it},$ the COVID-19 case count observed in week *t* in the neighbourhood *i* (*i* = 1,….*N* and *t* = 1,…..*T*), the data model takes Poisson distributions as the likelihood in Eq. (1):

1 $${y}_{it}\sim Poisson \left({\mu }_{it}\right)$$

Specifically, Poisson mean, ${\mu }_{it}$ is a product of ${n}_{i}$, the total number of populations in neighbourhood *i, o*btained from the 2016 census, is assumed to be time-variant*,* and ${\theta }_{it},$ the underlying unknown COVID-19 risk in the neighbourhood *i* during week *t*. The space–time variability is partitioned into three components: spatial, temporal and the space–time interaction effect. In Model 1, a space–time separable model is used that consists of the first two components (spatial, temporal), where the variability of data is not captured by the space–time separable structure. Models 2,3, and 4 capture the space–time inseparable structure proposed by Knorr-held^63^, using three different space–time interaction effects that allow space–time inseparability. Table 2 and appendix 3 (Supplementary Information 2) summarise the four Bayesian Space–Time Hierarchical models, including data, process, space–time interaction components, and full model specifications^31^.

**Table 2:** Summary of the four Bayesian space–time hierarchical models.

	Model 1	Model 2 Type I	Model 3 Type II	Model 4 Type III
Framework	Space–time separable	Space–time inseparable		
Data model	${y}_{it}\sim Poisson \left({\mu }_{it}\right), {\mu }_{it}= {n}_{i}. {\theta }_{it}$ where Poisson mean, ${\mu }_{it}$ is a product of ${n}_{i}$, the total number of populations in neighbourhood *i* is assumed to be time-varian*t,* and ${\theta }_{it},$ the underlying unknown COVID-19 rate in neighbourhood *i* during week *t*			
Process model	${\mathrm{log}(\theta }_{it})= \propto +beta1*{EDU}_{it}+beta2*{IMMI}_{it} +beta3*{LST}_{it} +\left({S}_{i}+{U}_{i} \right)+{v}_{t}$	${\mathrm{log}(\theta }_{it})= \propto +beta1*{EDU}_{it}+beta2*{IMMI}_{it}+beta3*{LST}_{it}+\left({S}_{i}+{U}_{i} \right)+{v}_{t}+ {\delta }_{it}$		
The overall spatial components	${S}_{1:140}\sim ICAR({W}_{sp},{\sigma }_{S}^{2})$ ${U}_{i}\sim N(0,{\sigma }_{U}^{2})$			
Overall temporal component	${v}_{1:89}\sim ICAR({W}_{RW1},{\sigma }_{v}^{2})$			
The space–time interaction component	${\delta }_{it}= 0$	${\delta }_{it}\sim N(0,{\sigma }_{\delta }^{2}$ )	${\delta }_{i,1:T }\sim RW({W}_{RW1},{\sigma }_{\delta }^{2})$	${\delta }_{i:N,t }\sim ICAR({W}_{SP},{\sigma }_{\delta }^{2})$
The parameter model	${\sigma }_{s} \sim Uniform\left(\mathrm{0.0001,10}\right), {\sigma }_{u} \sim Uniform\left(\mathrm{0.0001,10}\right),$ ${\sigma }_{v} \sim Uniform\left(\mathrm{0.0001,10}\right), {\sigma }_{\delta } \sim Uniform\left(\mathrm{0.0001,10}\right),$ $\propto \sim Uniform(-\infty ,+\infty )$			
Other vague priors	$beta1 \sim Normal\left(\mathrm{0,0.0001}\right)$, $beta2 \sim Normal\left(\mathrm{0,0.0001}\right)$, $beta3 \sim Normal\left(\mathrm{0,0.0001}\right)$			

where $beta1,beta2 \, and \, beta3$ are the regression coefficients of higher education rate, percentage of immigrants and LST, respectively.

${S}_{i}$ is the structured, ${U}_{i}$ is the unstructured, ${v}_{t}$ is the temporal and ${\delta }_{it}$ is the space–time interaction effect. ${\sigma }_{s}, {\sigma }_{u},{\sigma }_{v},{\sigma }_{\delta }$ are the standard deviation of the spatially-structured, spatially-unstructured, temporal and space–time random effect terms, respectively.

$1/{\sigma }_{s}^{2} , 1/{\sigma }_{u}^{2} ,1/{\sigma }_{v}^{2} 1/{\sigma }_{\delta }^{2}$ are the precision parameters.

*ICAR* Intrinsic Conditional Autoregressive, *RW* Random Walk Model.

### WinBUGS implementation

The space–time separable and the three space–time inseparable models were fitted with Markov Chain Monte Carlo (MCMC) with different initial values for each model with a burn-in period of 116,000 iterations and thinning rate of 10 to obtain the posterior distributions of model parameters using WinBUGS software version 1.4. Mixings were observed using trace plots and autocorrelation plots. Convergence was evaluated by checking the Gelman–Rubin statistic^35^ (Appendix 3 and Fig. S2 in Supplementary Information 2). After the burn-in period, a final run of 10,000 iterations for each chain was run to derive a final sample size of 20,000. The MCMC error of the model parameter estimates were < 5% of the corresponding posterior standard deviations suggesting that the total 20,000 iterations, 10,000 from each of the two MCMC chains, are sufficient to provide a good approximation of the posterior distribution.

### Model selection

We assessed the Deviance information criteria (*DIC*) and the probability of direction (*pD*) values from the outputs to evaluate the goodness of fit for the four Bayesian hierarchical models and to select the best-fitted model^64^.

### Spatial, temporal and spatiotemporal relative risks (RR)

The spatial, temporal and spatiotemporal relative risk estimates were obtained from the Model 3 (selected as the best-fitted model) outputs. Spatial relative risk (${RR}_{Spatial}=\mathrm{exp}({S}_{i}+{U}_{i}))$^38^ for neighbourhood *i* represents the average COVID-19 incidence rate over 89 weeks in neighbourhood *i* compared to the average COVID-19 incidence rate in Toronto. A map of the posterior means of the spatial relative risk was created using the ArcGIS Desktop software 10.8.1^46^. The temporal relative risk (${RR}_{Temporal}=\mathrm{exp}({v}_{t}))$^65^ at week *t* represents the average COVID-19 incidence rate for all neighbourhoods in week *i* compared to the average COVID-19 incidence rate of the entire study period. The posterior mean of the temporal relative risk with its corresponding 95% credible intervals was plotted using RStudio Software^60^ version 2021.09.0. The spatiotemporal effect term $\delta$ represents a change that cannot be reflected by spatial and temporal effects only^65^. The spatiotemporal relative risk ${(RR}_{SpatioTemporal}=\mathrm{exp}({\delta }_{it}))$^65^ represent the risk of COVID incidence rate in neighbourhood *i* and in time *t* compared to the overall incidence rate in entire study area and entire time period.

### Joinpoint regression

We used the joinpoint regression using the Joinpoint software version 4.9.0.0^66,67^, which uses the least-squares regression method to find the best-fit line from the temporal (weekly) pattern of the relative risk for COVID-19 derived from the Bayesian model. The joinpoint regression uses an algorithm that tests whether a multi-segmented line is a significantly better fit than a straight or less-segmented line. It involves fitting a series of joined straight lines on a log scale to the trends in the weekly relative risk of COVID-19. Line segments are joined at points called joinpoints. Each joinpoint denotes a statistically significant (P = 0.05) change in trend. The significance test uses a Monte Carlo Permutation method to find the best fit line for each segment. The temporal patterns of the relative risk was plotted using the JoinPoint software.

### Spatiotemporal risk trend analysis

The spatiotemporal trend of the relative risk over time (increasing, decreasing, or stable) in the neighbourhoods was calculated in RStudio Software^0^ and mapped in ArcGIS Desktop software^46^ version 10.8.1. The neighbourhoods with a negative estimated coefficient were considered to have a decreasing trend over time, and the neighbourhoods with positive estimated coefficients were considered to have an increasing trend over time. The neighbourhoods with zero estimated coefficients were considered to have a stable trend over the study period.

### Sensitivity analysis

Given that there is no such thing as the true prior^31^, two additional models with two alternate prior assumptions than the original final model (Model 3) were run to perform a sensitivity analysis to examine the final model results. The first model was run with hyperprior distributions of Gamma (0.005, 0.005)^68^ on the precision parameters ($1/{{\varvec{\sigma}}}_{{\varvec{s}}}^{2} , 1/{{\varvec{\sigma}}}_{{\varvec{u}}}^{2} ,1/{{\varvec{\sigma}}}_{{\varvec{v}}}^{2} 1/{{\varvec{\sigma}}}_{{\varvec{\delta}}}^{2}$), and the second model was run with a uniform prior with a wider range (0.0001,1000)^38^ for the ${{\varvec{\sigma}}}_{{\varvec{s}}},\boldsymbol{ }{{\varvec{\sigma}}}_{{\varvec{u}}},{{\varvec{\sigma}}}_{{\varvec{v}}},{{\varvec{\sigma}}}_{{\varvec{\delta}}}$ parameters (standard deviation of the spatially-structured, spatially-unstructured, temporal and space–time random effect term, respectively). The outputs from these two models with two different priors were compared to the outputs from the original model to ensure that our findings were not sensitive to the original hyperprior specification.

**Figure 2:**
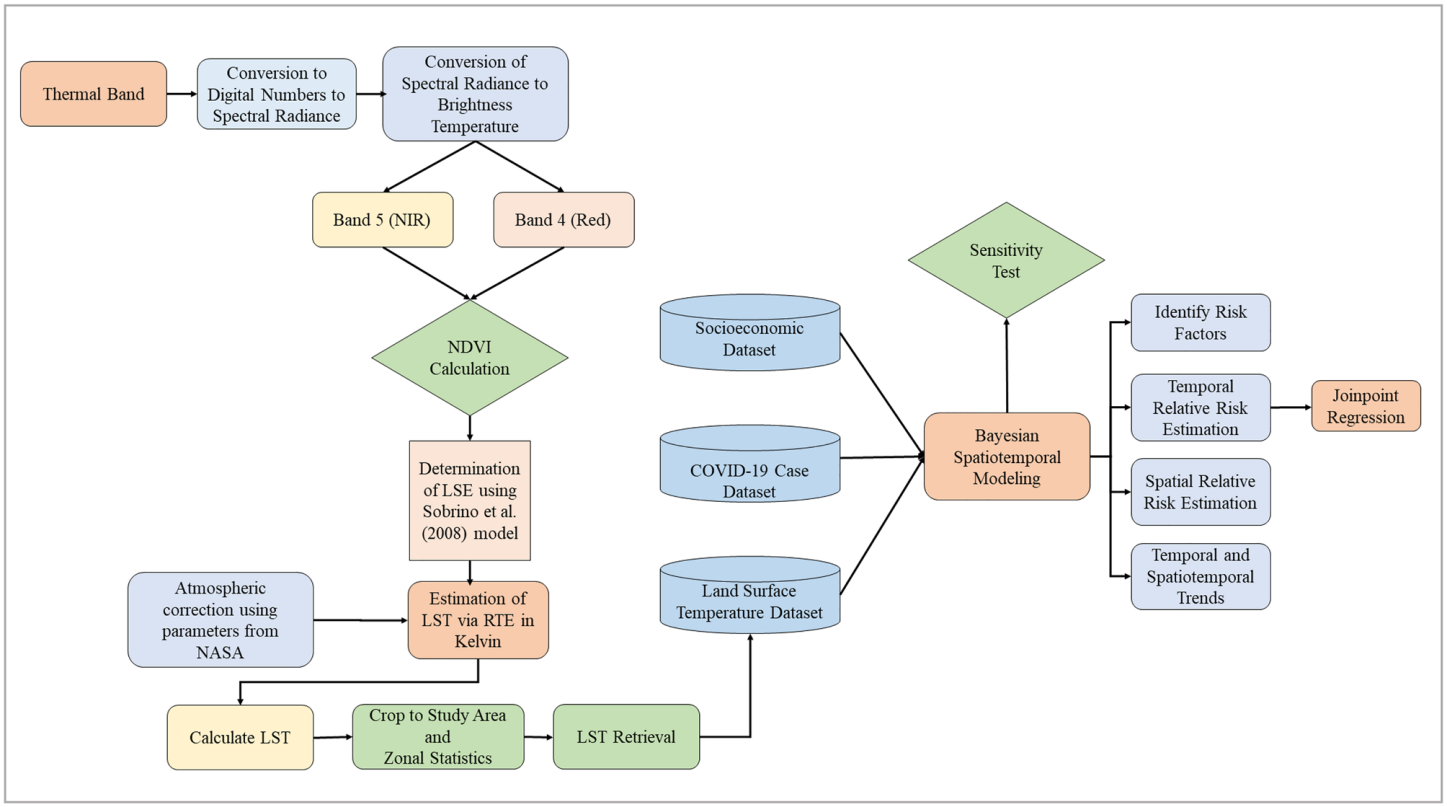
Flow diagram of the methodological framework.

The methodological framework for our study is shown in a flow diagram (Fig. 2).

## Results

### Descriptive statistics

We had 2,731,571 population in the study area based on the 2016 census data. A total of 179,072 (22,326 outbreak-associated and 156,746 sporadic) cases were reported in the 140 neighbourhoods during the study period. To avoid potential bias towards finding the high-risk clusters in the outbreak areas, our study excluded the outbreak-associated cases (12.4% of all cases), generally from healthcare (e.g., long-term care homes, hospitals) or congregated settings^7^. We also excluded 2,423 (1.64%) cases due to missing neighbourhood information, leaving 154,323 sporadic cases for the analysis.

**Figure 3:**
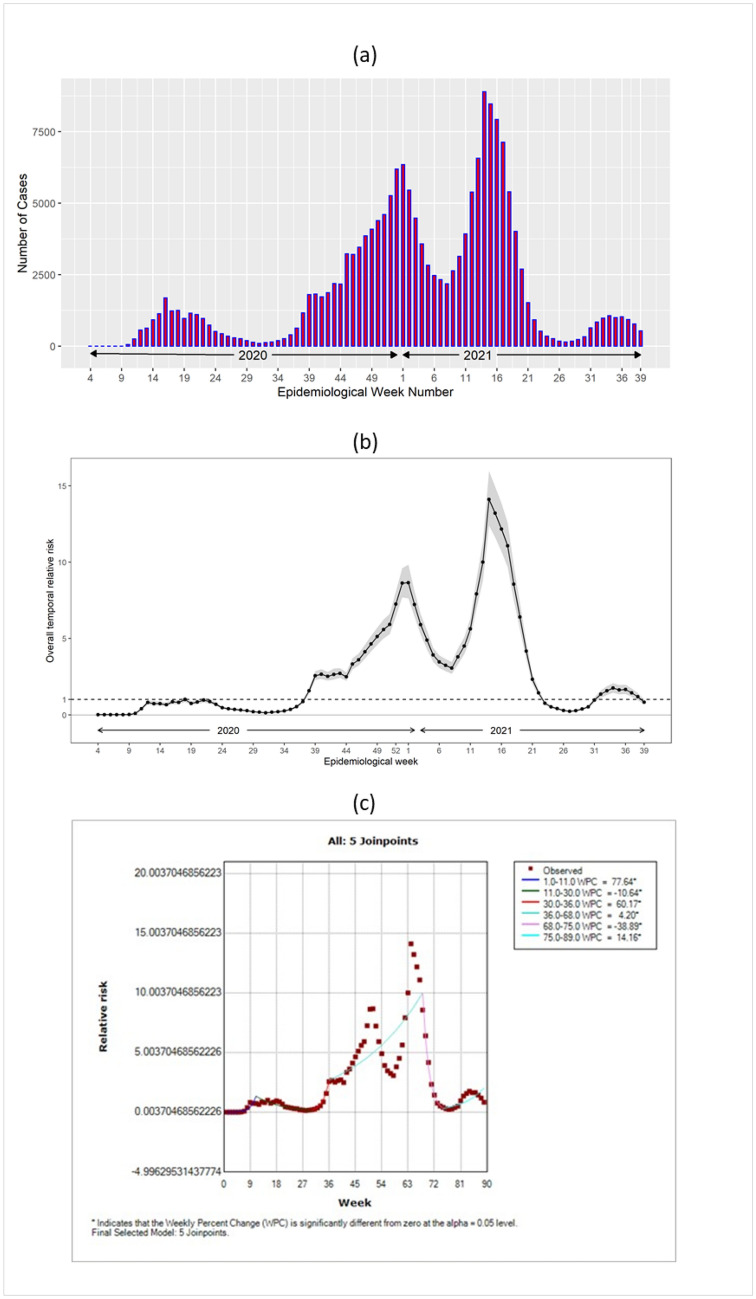
(**a**) The weekly number of COVID-19 cases (excluding the outbreak cases) between January 21, 2020, and October 2, 2021. (**b**) Temporal Relative Risk ((${RR}_{Temporal}=\mathrm{exp}({v}_{t}))$ of COVID-19 in Toronto neighbourhoods between January 21, 2020, and October 2, 2021. (**c**) The spatiotemporal trend of the relative risks ${(RR}_{SpatioTemporal}=\mathrm{exp}({\delta }_{it}))$ in Toronto between January 21, 2020, and October 2, 2021.

We observed a sharp increase in COVID-19 cases in late December of 2000, which declined in early January 2021 (Fig. 3a). Again, a sharp increase in the cases was observed in late March (week 13) of 2021, which declined in late May of the same year. The highest number of cases (> 7500 cases) were observed in April 2021.

### Model selection

We fitted the data in four Bayesian Space–Time Hierarchical Models and compared the models (Table 3). The *DIC* values from the space–time inseparable models (Model 2–4) are much lower than the space–time separable model (Model 1), suggesting that the inseparable models (Model 2, 3 and 4) are better supported by the observed data, confirming the need to incorporate the space–time interaction component^38^. Finally, by comparing Model 2, 3 and Model 4, we found that Model 3 has the smallest *pD*, and *DIC* values, suggesting that Model 3 (space–time inseparable model with type II interaction) is the most parsimonious, and therefore was selected as our final model.

**Table 3:** Comparison of the four space–time models.

Bayesian model	Space–time inseparable?	Space–time interaction type	*pD*	*DIC*
Model 1	No	NA	246.697	66,280.700
Model 2	Yes	Type I	5017.320	56,711.300
Model 3	Yes	Type II	3231.73	55,479.100
Model 4	Yes	Type III	4626.760	57,043.700

### Regression outputs

Between January 21, 2020, and October 2, 2021, using the parameter estimates (mean [${\delta }_{it}$] × 1000) from the final model, the average COVID-19 rate per 1000 population per neighbourhood in Toronto is estimated to be 6.1 (95% CI 6.0–6.3). Table 4 reports the estimated relative risks by exponentially transforming the regression covariates associated with the three covariates from the Model 3 regression outputs. Based on the outputs, we observed that higher education was negatively associated (95% CI 0.67–0.78), and LST (95% CI 1.01–1.17) was positively associated with COVID-19 incidence. An increase of one standard deviation in the higher education rate in a neighbourhood was associated with a 28% (95% CI 22–33%) standard deviation decrease in the COVID-19 incidence rate. An increase of one standard deviation in average LST in a neighbourhood was associated with a 9% (95% CI 1–17%) standard deviations increase in the COVID-19 incidence in Toronto. COVID-19 incidence was not found to be associated with the immigrants (95% CI 0.98–1.05), as the 95% CI contains 1. Therefore, the percentage of immigrants does not appear to be a strong risk factor for explaining variability in the COVID-19 incidence in our study (Table 4).

**Table 4:** Estimated relative risks [$\mathrm{exp}({\beta }_{k})]$ and 95% CI.

Relate Risk (RR)	Posterior Estimates of Risk (95% CI)
${\mathrm{RR}}_{\mathrm{EDU}}$ (Higher Education)	0.72 (0.67–0.78)
${\mathrm{RR}}_{\mathrm{IMMI}}$ (Immigrants)	1.02 (0.98–1.05)
${\mathrm{RR}}_{\mathrm{LST}}$ (LST)	1.09 (1.01–1.17)

### Temporal relative risk

Figure 3b presents the posterior mean and 95% uncertainty band of the temporal relative risk ((${RR}_{Temporal}=\mathrm{exp}({v}_{t}))$^65^ during the study period, which shows that the highest risk (RR > 9) was observed between March 14, 2021, and April 17, 2021. A total of 46 (51%) weeks out of the total 89 weeks experienced a relative risk of less than one during our study period.

### Joinpoint regression results

Figure 3c represents the temporal patterns of relative risk for COVID-19 from January 21, 2020, to October 2, 2021 (89 consecutive weeks). The line displays five joinpoints (6 line segments or trends), indicating a significant change in the relative risk six times during the study period. For instance, the relative risk of COVID-19 was increased by 77% per week from the beginning to the 11th week. The risk was then decreased by 11% by the 30th week, then was increased by 60% until the 36th week, then increased only by 4% until the 69th week, then it decreased by 39% until the 75th week, and it again increased by 14% by the end of the study period.

### Spatial relative risk

**Figure 4:**
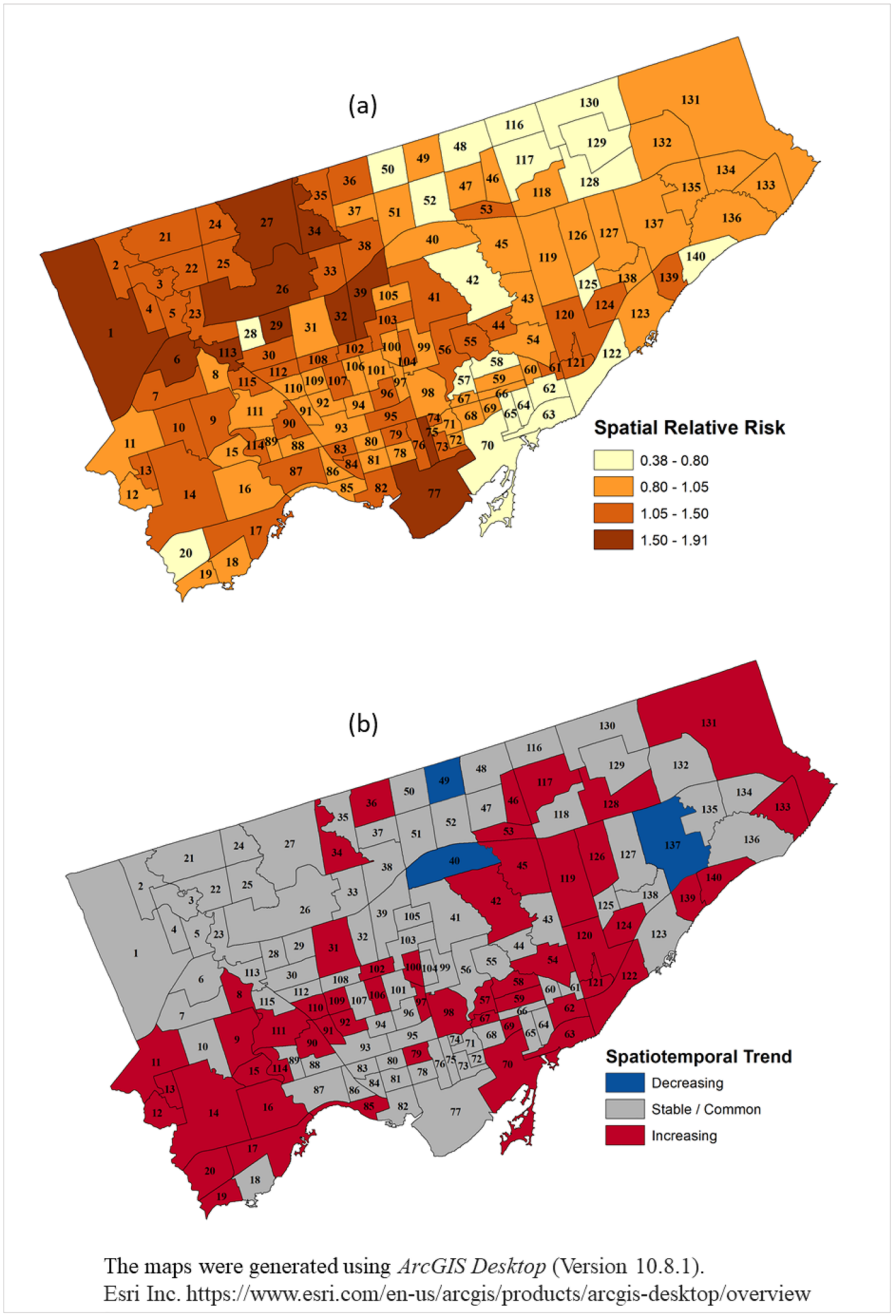
(**a**) A map of the estimated overall spatial pattern based on the posterior means of the spatial relative risks ${(RR}_{Spatial}=\mathrm{exp}({S}_{i}+{U}_{i}))$ for COVID-19 in the Toronto neighbourhoods, January 21, 2020–October 2, 2021. (**b**) The spatiotemporal trend of the relative risks ${(RR}_{SpatioTemporal}=\mathrm{exp}({\delta }_{it}))$ in Toronto between January 21, 2020, and October 2, 2021. The numbers inside the neighbourhood represents the neighbourhood identification number.

The posterior means of the relative risks (spatial) of COVID-19 in the Toronto neighbourhoods are presented in Fig. 4a. A high risk (RR > 1.5) was observed in northwestern and southern (Neighbourhood # 77) Toronto. A moderate level of risk (RR > 1.05) was observed in different neighbourhoods in western Toronto. Eastern Toronto mostly experienced a low risk of COVID-19.

### Spatiotemporal relative risk trends

While evaluating the trends of the spatiotemporal relative risks ${(RR}_{SpatioTemporal}=\mathrm{exp}({\delta }_{it}))$^65^ we observed that only three neighbourhoods (Neighbourhoods # 40,137 and 49) had a decreasing trend of the relative risk during the study period (Fig. 4b). A total of 84 (60%) neighbourhoods had a stable trend, and 53 (37%) neighbourhoods had an increasing trend in the study area.

### Sensitivity test results

The sensitivity test models with Gamma (0.005, 0.005)^68^ priors for the precision parameters and uniform (0.0001, 1000)^38^ priors for standard deviation parameters are equally appropriate, giving almost identical posterior distributions of the parameters and model fit (DIC values) compared to outputs of the original model (Model 3), presented in Appendix 4 in the Supplementary Information 2. Therefore, we can conclude that an apparently innocuous uniform prior that we have used in our final model is not introducing substantial information into the model fitting.

#### Map Validation

We mapped the spatial patterns of COVID-19 cases during the post-study period (October 3–31, 2021) to visually compare the spatial patterns of risk identified from the Bayesian spatiotemporal model (Fig. S3 in Appendix 5 in Supplementary Information 2). As observed, the spatial risk obtained from the Bayesian model was higher in the northwestern and southern neighbourhoods of Toronto. In these neighbourhoods, a higher number of cases were also reported during the post-study period. The map of the spatiotemporal trend showed an increasing pattern in the eastern, southwestern and central neighbourhoods. These neighbourhoods experienced a higher number of cases during the post-study period. In Northwestern Toronto, the trend was stable but observed a higher spatial risk, indicating that the area remained at higher risk throughout the study period. This region also showed a higher number of cases during the post-study period.

## Discussion

In our study, we observed spatial, temporal, and spatiotemporal trends of COVID-19 in Toronto and identified the key factors associated with the transmission of the disease. Overall, the trend and transmission patterns of the disease were heterogeneous over space and time. Only three neighbourhoods experienced a decreasing spatiotemporal risk trend in the area. Most neighbourhoods experienced either stable or increasing spatiotemporal risk during the study period. We also observed several high-risk neighbourhoods in the western and southern parts of Toronto, and the risk in those neighbourhoods remained constant throughout the study period. Since educational status and LST were associated with the risk, we believe these factors might have influenced to remain those areas at high risk throughout the study period.

The temporal risk was particularly high in the early spring of 2021, suggesting that the temperature during the season could have influenced the transmission of the disease in that part of the country. However, some other factors, such as an increase in mobility or travel patterns during the long weekend in March 2021^69^, could also influence the increase of the disease in spring. The disease risk remained high for more than half of the study period. We observed a lower risk during early summer (June–July) of 2020 and 2021, which could be due to an increased time spent in outdoor settings leading to a decreased COVID-19 risk^70^. We identified significant changes in the risk of COVID-19 six times across the study period with varying trends. These changes suggest that the temporal trend of the epidemic of COVID-19 is different from other coronavirus diseases, such as the SARS (severe acute respiratory syndrome) epidemic in 2003, where the number of reported SARS cases has increased exponentially over time, and the outbreak lasted approximately 6 months^71–74^.

The findings of the positive association of COVID-19 with LST in our study are consistent with the findings of an earlier study^9^. One reason behind this could be that high LST favours the coronavirus. Furthermore, Toronto is the most densely populated urban city in Canada. A recent study conducted in Phoenix, Arizona and Dallas, Texas, in the United States by Moss and Kar^75^ concluded that the urban areas that are susceptible to a high Urban Heat Index, as measured by LST, are primarily occupied by vulnerable population groups. Therefore, it is possible that an excess vulnerable population in an area creates a higher LST in the neighbourhood, resulting in an increase in the risk of COVID-19 in our study area.

Our study also finds that the higher education rate has a negative association with COVID-19, which is in line with the findings from earlier studies^16,19,21,22^. It is likely that people with higher education may understand and follow public health messages as well as have the option to work remotely and maintain social distancing, resulting in lower incidences of COVID-19 in areas with a higher number of educated people. Additionally, a study by Mondal et al. in 2021 found that higher education levels were associated with a higher likelihood of vaccine acceptance^76^, suggesting that intervention with health education may play a key role in fighting this pandemic. Various public health programs such as COVID-19 awareness and health education programs in neighbourhoods with low education may also help reduce the fast transmission rates in those neighbourhoods.

In our study, we used higher spatial and temporal resolution satellite images to extract LST. We also used atmospheric corrections methods on these images by adopting Sobrino et al. in 2008’s Land Surface Emissivity (LSE) model^77^, which provided a high estimation accuracy. Our findings may advocate for maintaining disease surveillance and planning for an effective public health program. Most of the earlier studies^11,78–82^ explored the relationship between ambient temperature or LST and COVID-19 at a broader spatial scale (provincial or state) and used a shorter period with a very limited number of images to extract LST without any atmospheric corrections^13^. We believe that our study filled the gap in the existing literature by using higher spatial and temporal resolution satellite imageries at a local spatial scale, which is more spatially representative and may have provided a more accurate estimate due to the use of the atmospherically corrected data on LST.

The validation results of the spatiotemporal patterns of risk using the data of the post-study period suggest that the Bayesian model could predict spatial patterns of risk for COVID-19 in our study area. Therefore, the findings of our study can be useful for increasing awareness of the disease and preparing public health interventions aimed at targeted prevention and control of COVID-19. Given limited resources available, efforts could focus on the high-risk neighbourhoods, as observed in our study.

Our study has several limitations. First, we could not find data on air pollution or human mobility patterns at the neighbourhood level, which could be important contributors to influencing the COVID-19 incidence. Second, we had 1.6% of cases with missing neighbourhood information and 12.4% cases were outbreak cases, which were not included in our analysis. Additionally, COVID-19 is often asymptomatic, under-reported^83^ or lacks accurate information on the onset of the COVID-19, limiting the capacity of the analysis. However, the Bayesian spatiotemporal hierarchical models allowed us to compensate for the missing/unobserved covariates or missing data by incorporating the structured, unstructured random effects into the model^63^. In particular, the Type II space–time interaction in our final model implied that the missing covariates have smoothly varying structures through time and have no structure over space since they are highly localized in their effect on the outcome^63^. Third, our study has an ecological study design where the data were aggregated at the neighbourhood level, which may create issues such as ecological fallacy^84^. Therefore, these results cannot be interpreted at the individual level.

Despite these limitations, our study, due to its strong versatility and complex hierarchical modelling, is still convincing and has provided important information that may improve our understanding of the transmission patterns of COVID-19 and the associated risk factors. Also, our model is superior to the frequentist method that is more frequently used, as the Bayesian approach allowed us to compensate for the missing covariates in the models in identifying spatial patterns of risk. Therefore, we believe that using the Bayesian spatiotemporal model and the long-time series satellite-derived environmental data for modelling disease transmission have advanced our understanding of the disease risk in space.

## Conclusions

Several conclusions can emerge from our study. First, the Bayesian analysis has shown that Bayesian regression with spatial (structured and unstructured), temporal and spatiotemporal random effects provided an effective framework for understanding COVID-19 disease transmission. Second, the spatiotemporal risk remained high for the entire study period and constantly high for the high-risk neighbourhoods. However, the temporal risk fluctuated over time in the study area. Third, higher education and LST played an important role in predicting COVID-19 incidence. Therefore, it is important to take those factors into account while planning intervention strategies. Fourth, the framework presented in this study may help make an early warning system for COVID-19 incidence and assist public health authorities in controlling and preventing outbreaks of similar diseases. Finally, the methodological framework applied here can also be used in other small area-level studies on infectious diseases.

## Supplementary Information

Supplementary Information 1.

Supplementary Information 2.

## Data Availability

All data generated or analysed during this study are included in this published article (and its supplementary information files).

## Abbreviations

DIC: Deviance information criteria
LSE: Land surface emissivity
LST: Land surface temperature
MCMC: Markov Chain Monte Carlo
NASA: National Aeronautics and Space Administration
NDVI: Normalized Difference Vegetation Index
NIR: Near infrared
pD: The probability of direction
RR: Relative risk
RTE: Radiative transfer equation
SARS: Severe acute respiratory syndrome
TIR: Thermal infra-red
TOA: Top of atmosphere
USGS: United States Geological Survey
