# Supplementary material for: Identifying spatiotemporal patterns of COVID-19 transmissions and the drivers of the patterns in Toronto: a Bayesian hierarchical spatiotemporal modelling
Source: Sci Rep. 2022 Jun 7;12:9369. doi: 10.1038/s41598-022-13403-x (PMC9172088; doi:10.1038/s41598-022-13403-x)
Supplement: Supplementary file 2 — Supplementary Information 2. [file 41598_2022_13403_MOESM2_ESM.docx]

Supplementary Information (Five Appendices) for:

**Identifying Spatiotemporal Patterns of COVID-19 Transmissions and the Drivers of the Patterns in Toronto: A Bayesian Hierarchical Spatiotemporal Modelling**

**Appendix 1: Land Surface Temperature (LST) Retrieval Method**

**Landsat Mission**

Landsat program, hosted by the USGS Earth Resources Observation and Science (EROS) centre, is the longest-running enterprise for the acquisition of satellite imagery of Earth^1^. For our study, we have used Landsat Collection 2 level 1 dataset. Landsat 8, the most recently launched Landsat satellite, has two sensors: the Operational Land Imager (OLI) and the Thermal Infrared (TIR). Landsat 8 and Landsat 7 satellites have a 16-day repeat cycle and circle the earth in a sun-synchronous, near-polar orbit, at an altitude of 705 km, inclined at 98.2 degrees. The satellites circles the earth orbit every 99 minutes with an equatorial crossing time: 10:00 AM ±15 minutes^2^. **Landsat 8** images consist of nine spectral bands with a spatial resolution of 30 meters for Bands 1 to 7 and 9, and 10 and 11 (thermal) are collected at 100 meters^1^. Landsat 7 images consist of eight spectral bands with a spatial resolution of 30 meters for Bands 1 to 7, while Band 6 (thermal) collects both high and low gain (60/30 meters) for all scenes.

**Land Surface Temperature (LST) Retrieval Method**

In the last few decades, several methodologies with improved algorithms for computing land surface temperature using satellite-based thermal infra-red (TIR) data has been developed^3–7^. A few studies comparing these methods found that Sobrino et al. 2008's Land Surface Emissivity (LSE) model^8^ provided the highest accuracy for extracting land surface temperature using the Landsat imagery^9–11^. We applied an automated extraction toolbox in ArcGIS Desktop developed by Aliiahsan et.al^9^; that computes Land Surface Temperature (LST) using the Radiative Transfer Equation (RTE) method based on Sobrino's LSE model^8^.

#### NDVI Threshold (NDVI^THM^)-Based LSE Model

We used the NDVI Thresholds method proposed by Sobrino et al. in 2008^8^ to compute the Land Surface Emissivity (LSE) from NDVI threshold values considering three different cases:1) NDVI <0.2, the pixel is considered as bare soil, and the emissivity is obtained from the reflectance values in the red region, 2) $0.2 \leq NDVI \geq0.5$, the pixel is composed of a mixture of bare soil and vegetation, 3) NDVI > 0.5, the pixels with NDVI values higher than 0.5 are considered as fully vegetated areas^8^. The model is expressed in the following equation:

$$\varepsilon= \left\{ \begin{matrix} {a_{i}\rho}_{R}+ b_{i} & NDVI<0.2 \\ \varepsilon_{v}+ \varepsilon_{v}\left( 1-P_{v} \right)+d\varepsilon, d\varepsilon=\left( 1-\varepsilon_{s} \right)\left( 1-P_{v} \right)F\varepsilon_{v} & 0.2 \leq NDVI \geq0.5 \\ \varepsilon_{v}+ d\varepsilon& NDVI>0.5 \end{matrix} \right\}$$

$\varepsilon_{v}$ is the soil and $\varepsilon_{s}$ is the vegetation emissivity. $d\varepsilon$ is the cavity effect due to surface roughness, *F* is a geometrical shape factor assumed as the mean value of 0.55^12^, $\rho_{R}$ is the reflectance value of the red band, $a_{i}$i and $b_{i}$ are estimated from an empirical relationship between the red band reflectance and Moderate Resolution Imaging Spectroradiometer (MODIS) emissivity library.

**LST calculation using radiative transfer equation (RTE)**

For the RTE-based method, we have used an atmospheric correction parameter calculator^13^ to extract global atmospheric profiles for each image date, time and location. The correction parameter used the NCEP (National Centre for Environmental Prediction) to simulate atmospheric transmittance, upwelling and downwelling radiances^7^. Removing the effects of the atmosphere in the thermal region is the essential step necessary to use the thermal band as atmospheric correction allows avoiding systematic errors in the predicted surface temperature^7^. With these parameters, the space-reaching radiance was converted to a surface-leaving radiance^7^.

$$L_{TOA}= {\tau\varepsilon L}_{T}+ L_{u}+(1-\varepsilon)L_{d}$$

where $\tau$ is the atmospheric transmission, $\varepsilon$ is the emissivity of the surface, $L_{T}$ is the radiance of a blackbody target of kinetic temperature $T$, $L_{u}$ is the upwelling or atmospheric path radiance, $L_{d}$ is the downwelling or sky radiance, and $L_{TOA}$ is TOA radiance measured by the instrument.

LST was retrieved using a single TIR band with the inversion of the radiative transfer equation (RTE) according to the following equation where $L_{\lambda}^{sen}$ (W∙m^−2^∙sr^−1^∙μm^−1^) is at-sensor registered radiance of the related thermal band, $B_{\lambda}$ (W∙m^−2^∙sr^−1^∙μm^−1^) is the blackbody radiance.

$$L_{\lambda}^{sen}=\left[ \varepsilon{B_{\lambda}(T}_{S} \right)+(1-\varepsilon)L_{\lambda}^{\downarrow}]\tau+L_{\lambda}^{\uparrow}$$

Blackbody radiance ($B_{\lambda})$ at a temperature of can be obtained by inverting the previous equation:

$${B_{\lambda}(T}_{S})=\frac{L_{\lambda}^{sen}-L_{\lambda}^{\uparrow}-\tau(1-\varepsilon)L_{\lambda}^{\downarrow}}{\tau\varepsilon}$$

Land Surface Temperature $T_{S}$ can be obtained by inverting Planck's Law as:

$$T_{S}=\frac{K_{2}}{ln\left[ \frac{K_{1}}{\frac{L_{\lambda}^{sen}-L_{\lambda}^{\uparrow}-\tau(1-\varepsilon)L_{\lambda}^{\downarrow}}{\tau\varepsilon}}+1 \right]}$$

#### where $\boldsymbol{K}_{\boldsymbol{1}}$ and $\boldsymbol{K}_{\boldsymbol{2}}$ are calibration constants for Landsat data reported.

**Retrieval of spectral radiance and Brightness temperature (*T*) retrieval**

The Top of Atmosphere (TOA) spectral radiance value is calculated for Landsat 7 using the following equation:

$$L_{\lambda}= \left[ \frac{{LMAX}_{\lambda}- {LMIN}_{\lambda}}{QCALMAX-QCALMIN} \right] x \left[ Q_{CAL}- QCALMIN \right]+ {LMIN}_{\lambda}$$

where $L_{\lambda}$ is TOA spectral radiance (Watts/(m^2^∙srad∙μm)), $Q_{CAL}$ is the quantized calibrated pixel value in DN, ${LMIN}_{\lambda}$ (Watts/(m^2^∙srad∙μm)) is the spectral radiance scaled to $QCALMIN$, ${LMAX}_{\lambda}$ (Watts/(m^2^∙srad∙μm)) is the spectral radiance scaled to $QCALMAX$, $QCALMIN$ is the minimum quantized calibrated pixel value in DN and $QCALMIN$ is the maximum quantized calibrated pixel value in DN. ${LMIN}_{\lambda}$, ${LMAX}_{\lambda}$, $QCALMIN$, and $QCALMAX$ values are obtained from the metadata file of Landsat 7 data^9^.

The TOA spectral radiance value is calculated for Landsat 8 using the following equation.

$$L_{\lambda}= M_{L} . Q_{CAL}+ A_{L}$$

where $L_{\lambda}$ is the TOA spectral radiance (Watts/(m^2^∙srad∙μm)),  $M_{L}$ is the band-specific multiplicative rescaling factor from the metadata, $A_{L}$ is the band-specific additive rescaling factor from the metadata, $Q_{CAL}$ is the quantized and calibrated standard product pixel values (DN).

The brightness temperature T can be generated by the following equation:

$$T= \frac{K_{2}}{ln(\frac{K_{1}}{L}+1)}$$

where T refers to the effective at-satellite brightness temperature in Kelvin, $K_{1}$ (Watts/(m^2^∙srad∙μm)) and $K_{2}$ (Kelvin) are the calibration constants and $L_{\lambda}$is the spectral radiance.

**Table S1: Parameters for the Land Surface Temperature (LST) calculation.**

|  |  |  |  |  | Atmospheric Correction Parameter Calculator (https://atmcorr.gsfc.nasa.gov/cgi-bin/atm_corr.pl) | | |  | |
| --- | --- | --- | --- | --- | --- | --- | --- | --- | --- |
| Week | **Date of image** | **Scene Center Time (GMT)** | **Landsat** | **Sun Elevation (From Metadata)** | **Atmospheric Transmission** | **Upwelling radiance** | **Downwelling radiance** | **Solar Zenith Angle**  **(For Landsat 7)** | **Earth-Sun Distance (From metadata)** |
| 1 | 24-Jan-20 | 16:03:55 | Landsat 8 | 24.35807661 | 0.91 | 0.54 | 0.91 |  |  |
| 2 | 01-Feb-20 | 15:43:06 | Landsat 7 | 24.49170863 | 0.92 | 0.38 | 0.64 | 65.5082914 | 0.9853188 |
| 3 | 02-Feb-20 | 15:57:41 | Landsat 8 | 26.50548037 | 0.92 | 0.43 | 0.74 |  |  |
| 4 | 09-Feb-20 | 16:03:50 | Landsat 8 | 28.49063776 | 0.94 | 0.29 | 0.5 |  |  |
| 5 | 18-Feb-20 | 15:57:38 | Landsat 8 | 31.37765848 | 0.91 | 0.52 | 0.88 |  |  |
| 6 | 25-Feb-20 | 16:03:47 | Landsat 8 | 33.8380565 | 0.89 | 0.59 | 1 |  |  |
| 7 | 05-Mar-20 | 15:57:32 | Landsat 8 | 37.19903778 | 0.96 | 0.21 | 0.37 |  |  |
| 8 | 12-Mar-20 | 16:03:40 | Landsat 8 | 39.91607 | 0.91 | 0.55 | 0.93 |  |  |
| 9 | 21-Mar-20 | 15:57:25 | Landsat 8 | 43.46096552 | 0.98 | 0.1 | 0.17 |  |  |
| 10 | 28-Mar-20 | 16:03:31 | Landsat 8 | 46.2074895 | 0.79 | 1.36 | 2.2 |  |  |
| 11 | 29-Mar-20 | 15:33:52 | Landsat 7 | 44.12493545 | 0.69 | 2.15 | 3.35 | 45.8750646 | 0.9985865 |
| 12 | 06-Apr-20 | 15:57:16 | Landsat 8 | 49.64471163 | 0.94 | 0.34 | 0.59 |  |  |
| 13 | 13-Apr-20 | 16:03:24 | Landsat 8 | 52.19555246 | 0.74 | 1.73 | 2.79 |  |  |
| 14 | 22-Apr-20 | 15:57:09 | Landsat 8 | 55.24010206 | 0.97 | 0.15 | 0.26 |  |  |
| 15 | 29-Apr-20 | 16:03:16 | Landsat 8 | 57.37985022 | 0.76 | 1.62 | 2.63 |  |  |
| 16 | 08-May-20 | 15:57:00 | Landsat 8 | 59.7895888 | 0.96 | 0.22 | 0.38 |  |  |
| 17 | 15-May-20 | 16:03:12 | Landsat 8 | 61.35021213 | 0.63 | 2.55 | 3.97 |  |  |
| 18 | 23-May-20 | 15:36:46 | Landsat 7 | 59.21636685 | 0.7 | 2.15 | 3.4 | 30.7836332 | 1.0126091 |
| 19 | 24-May-20 | 15:57:03 | Landsat 8 | 62.90624602 | 0.63 | 2.8 | 4.36 |  |  |
| 20 | 31-May-20 | 16:03:17 | Landsat 8 | 63.75394942 | 0.92 | 0.52 | 0.9 |  |  |
| 21 | 09-Jun-20 | 15:57:12 | Landsat 8 | 64.37367127 | 0.71 | 2.37 | 3.79 |  |  |
| 22 | 16-Jun-20 | 16:03:27 | Landsat 8 | 64.50220934 | 0.86 | 1.05 | 1.76 |  |  |
| 23 | 25-Jun-20 | 15:57:21 | Landsat 8 | 64.24295184 | 0.78 | 1.58 | 2.62 |  |  |
| 24 | 02-Jul-20 | 16:03:35 | Landsat 8 | 63.74261212 | 0.65 | 2.83 | 4.48 |  |  |
| 25 | 11-Jul-20 | 15:57:28 | Landsat 8 | 62.75393472 | 0.51 | 3.78 | 5.7 |  |  |
| 26 | 18-Jul-20 | 16:03:41 | Landsat 8 | 61.73733148 | 0.64 | 2.99 | 4.68 |  |  |
| 27 | *Data Unavailable* | | | | | | | | |
| 28 | 27-Jul-20 | 15:57:32 | Landsat 8 | 60.14323089 | 0.47 | 4.33 | 6.37 |  |  |
| 29 | 03-Aug-20 | 16:03:45 | Landsat 8 | 58.6961457 | 0.63 | 2.82 | 4.4 |  |  |
| 30 | 12-Aug-20 | 15:57:36 | Landsat 8 | 56.58795429 | 0.83 | 1.36 | 2.27 |  |  |
| 31 | 19-Aug-20 | 16:03:50 | Landsat 8 | 54.76228155 | 0.83 | 1.22 | 2.02 |  |  |
| 32 | 28-Aug-20 | 15:57:44 | Landsat 8 | 52.2044129 | 0.7 | 2.32 | 3.68 |  |  |
| 33 | 04-Sep-20 | 16:03:58 | Landsat 8 | 50.06588993 | 0.83 | 1.2 | 2 |  |  |
| 34 | 12-Sep-20 | 15:29:52 | Landsat 7 | 44.03172077 | 0.72 | 1.9 | 3.04 | 45.9682792 | 1.0062831 |
| 35 | 13-Sep-20 | 15:57:51 | Landsat 8 | 47.1568631 | 0.69 | 2.42 | 3.82 |  |  |
| 36 | 20-Sep-20 | 16:04:04 | Landsat 8 | 44.79112835 | 0.9 | 0.67 | 1.13 |  |  |
| 37 | 29-Sep-20 | 15:57:55 | Landsat 8 | 41.67289377 | 0.84 | 1.13 | 1.89 |  |  |
| 38 | 06-Oct-20 | 16:04:07 | Landsat 8 | 39.21883602 | 0.79 | 1.4 | 2.3 |  |  |
| 39 | 15-Oct-20 | 15:57:57 | Landsat 8 | 36.08716502 | 0.78 | 1.43 | 2.35 |  |  |
| 40 | 22-Oct-20 | 16:04:07 | Landsat 8 | 33.70881838 | 0.71 | 1.98 | 3.13 |  |  |
| 41 | 31-Oct-20 | 15:57:56 | Landsat 8 | 30.80408106 | 0.95 | 0.27 | 0.47 |  |  |
| 42 | *Data Unavailable* | | | | | | | | |
| 43 | 08-Nov-20 | 15:19:57 | Landsat 7 | 25.8407203 | 0.89 | 0.83 | 1.37 | 64.1592797 | 0.9906193 |
| 44 | 16-Nov-20 | 15:57:54 | Landsat 8 | 26.28530259 | 0.94 | 0.34 | 0.59 |  |  |
| 45 | 23-Nov-20 | 16:04:07 | Landsat 8 | 24.66576502 | 0.92 | 0.44 | 0.76 |  |  |
| 46 | 02-Dec-20 | 15:57:58 | Landsat 8 | 22.99062118 | 0.96 | 0.21 | 0.36 |  |  |
| 47 | 09-Dec-20 | 16:04:09 | Landsat 8 | 22.03707882 | 0.9 | 0.54 | 0.92 |  |  |
| 48 | 18-Dec-20 | 15:57:57 | Landsat 8 | 21.30569479 | 0.96 | 0.19 | 0.33 |  |  |
| 49 | 25-Dec-20 | 16:04:06 | Landsat 8 | 21.14419286 | 0.94 | 0.31 | 0.52 |  |  |
| 50 | 02-Jan-21 | 15:22:10 | Landsat 7 | 18.13418977 | 0.92 | 0.48 | 0.79 | 71.8658102 | 0.9833016 |
| 51 | 03-Jan-21 | 15:57:52 | Landsat 8 | 21.47295072 | 0.89 | 0.6 | 1.02 |  |  |
| 52 | 10-Jan-21 | 16:04:00 | Landsat 8 | 22.14366309 | 0.95 | 0.27 | 0.47 |  |  |
| 53 | 19-Jan-21 | 15:57:45 | Landsat 8 | 22.14366309 | 0.95 | 0.22 | 0.39 |  |  |
| 54 | 26-Jan-21 | 16:03:56 | Landsat 8 | 24.96601689 | 0.91 | 0.46 | 0.78 |  |  |
| 55 | 04-Feb-21 | 15:57:44 | Landsat 8 | 27.25849939 | 0.96 | 0.18 | 0.32 |  |  |
| 56 | 11-Feb-21 | 16:03:53 | Landsat 8 | 29.34081589 | 0.97 | 0.12 | 0.21 |  |  |
| 57 | 20-Feb-21 | 15:57:39 | Landsat 8 | 32.3300296 | 0.97 | 0.16 | 0.28 |  |  |
| 58 | *Data Unavailable* | | | | | | | | |
| 59 | 28-Feb-21 | 15:11:48 | Landsat 7 | 30.49313654 | 0.87 | 0.75 | 1.25 | 59.5068635 | 0.9906971 |
| 60 | 08-Mar-21 | 16:04:09 | Landsat 8 | 22.03707882 | 0.91 | 0.52 | 0.88 |  |  |
| 61 | 15-Mar-21 | 16:03:38 | Landsat 8 | 41.00473898 | 0.97 | 0.14 | 0.24 |  |  |
| 62 | 24-Mar-21 | 15:57:25 | Landsat 8 | 44.55284896 | 0.88 | 0.7 | 1.18 | 45.447151 | 1.0058369 |
| 63 | 31-Mar-21 | 16:03:34 | Landsat 8 | 47.28224338 | 0.87 | 0.76 | 1.27 |  |  |
| 64 | 09-Apr-21 | 15:57:20 | Landsat 8 | 50.67232025 | 0.74 | 1.8 | 2.89 |  |  |
| 65 | 16-Apr-21 | 16:03:28 | Landsat 8 | 53.16767185 | 0.87 | 0.74 | 1.24 |  |  |
| 66 | 24-Apr-21 | 15:13:30 | Landsat 7 | 49.52985399 | 0.88 | 0.7 | 1.18 | 40.470146 | 1.0058369 |
| 67 | 25-Apr-21 | 15:57:13 | Landsat 8 | 56.11881991 | 0.92 | 0.49 | 0.83 |  |  |
| 68 | 02-May-21 | 16:03:19 | Landsat 8 | 58.16904696 | 0.74 | 1.75 | 2.84 |  |  |
| 69 | 11-May-21 | 15:57:09 | Landsat 8 | 60.45369328 | 0.92 | 0.44 | 0.76 |  |  |
| 70 | 18-May-21 | 16:03:25 | Landsat 8 | 61.89629835 | 0.81 | 1.45 | 2.41 |  |  |
| 71 | 27-May-21 | 15:57:19 | Landsat 8 | 63.29413436 | 0.9 | 0.62 | 1.07 |  |  |
| 72 | 03-Jun-21 | 16:03:33 | Landsat 8 | 64.01577194 | 0.67 | 2.44 | 3.83 |  |  |
| 73 | 12-Jun-21 | 15:57:26 | Landsat 8 | 64.47412564 | 0.72 | 2.14 | 3.42 |  |  |
| 74 | 19-Jun-21 | 16:03:39 | Landsat 8 | 64.48534021 | 0.76 | 1.88 | 3.09 |  |  |
| 75 | 20-Jun-21 | 15:02:34 | Landsat 7 | 56.08455683 | 0.69 | 2.26 | 3.55 | 33.9154432 | 1.0162019 |
| 76 | 28-Jun-21 | 15:57:30 | Landsat 8 | 64.09149511 | 0.52 | 3.79 | 5.72 |  |  |
| 77 | 05-Jul-21 | 16:03:42 | Landsat 8 | 63.49402232 | 0.62 | 3.07 | 4.77 |  |  |
| 78 | 14-Jul-21 | 15:57:31 | Landsat 8 | 62.38916443 | 0.67 | 2.67 | 4.19 |  |  |
| 79 | 21-Jul-21 | 16:03:45 | Landsat 8 | 61.29353932 | 0.84 | 1.19 | 1.99 |  |  |
| 80 | 30-Jul-21 | 15:57:39 | Landsat 8 | 59.60748559 | 0.86 | 0.98 | 1.65 |  |  |
| 81 | 06-Aug-21 | 16:03:53 | Landsat 8 | 58.08933179 | 0.72 | 2.24 | 3.61 |  |  |
| 82 | 14-Aug-21 | 15:04:00 | Landsat 7 | 48.01591965 | 0.78 | 1.57 | 2.54 | 41.9840804 | 1.0129154 |
| 83 | 15-Aug-21 | 15:57:45 | Landsat 8 | 55.89414317 | 0.84 | 1.26 | 2.1 |  |  |
| 84 | 22-Aug-21 | 16:03:58 | Landsat 8 | 54.01089576 | 0.53 | 3.66 | 5.59 |  |  |
| 85 | 31-Aug-21 | 15:57:50 | Landsat 8 | 51.38469741 | 0.77 | 1.75 | 2.84 |  |  |
| 86 | 07-Sep-21 | 16:04:03 | Landsat 8 | 49.19662115 | 0.79 | 1.62 | 2.66 |  |  |
| 87 | 16-Sep-21 | 15:57:54 | Landsat 8 | 46.23729378 | 0.79 | 1.58 | 2.6 |  |  |
| 88 | 23-Sep-21 | 16:04:05 | Landsat 8 | 43.84762947 | 0.77 | 1.66 | 2.68 |  |  |
| 89 | 02-Oct-21 | 15:57:59 | Landsat 8 | 40.7113483 | 0.74 | 1.99 | 3.22 |  |  |

**Appendix 2**

**Fig. S1: Correlation Matrix of the independent variables**.


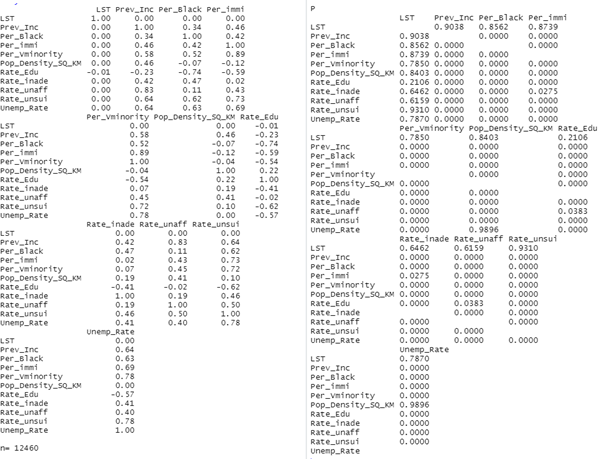


**Table S2**: Inclusion Probabilities from the BayesVarSel Method.

Incl.prob. HPM MPM

LST 1 * *

Prev_Inc 0.038

Per_immi 1 * *

Pop_Density_SQ_KM 0.0312

Rate_Edu 1 * *

Rate_inade 0.0318

**Appendix 3**

**Spatiotemporal Model Specifications**

Prior distributions were assigned for all model parameters. A vague uniform prior (0, 0001, 1000) was assigned independently to the standard deviations of the spatial, temporal and space-time random effect terms $\boldsymbol{\sigma}_{\boldsymbol{s}}\boldsymbol{,}\boldsymbol{\sigma}_{\boldsymbol{u}}\boldsymbol{,}\boldsymbol{\sigma}_{\boldsymbol{v}}\boldsymbol{,}\boldsymbol{\sigma}_{\boldsymbol{\delta}}$, respectively. The intercept $\propto$ has the improper uniform prior. The overall spatial random effect component was modelled using the Besag York Mollié (BYM model)^32^. The BYM model is a convulsion of a spatially structured random effect and a spatially unstructured random effect following a Gaussian distribution (Table 2). The conditional autoregressive (CAR) prior with a spatial adjacency matrix *W* of Size *N x N*, where $w_{ij}=0$, and the off-diagonal entries $w_{ij}=1$, if areas *i* and *j* share a common boundary lines and otherwise^33^. The CAR prior on the spatial random effects implies that adjacent neighbourhoods tend to have similar overall COVID-19 risks. The overall temporal component was modelled using the RW1 model Fahrmeir and Lang^34^ (2001) to describe the overall time trend common to all neighbourhoods. The RW1 is a temporal adaptive process that represents a one-dimensional analogue of the ICAR, and the parameter at the first time point *t*, is assigned a vague/diffuse prior. A noninformative prior $Normal\left( 0,0.0001 \right)$ is assigned to the three regression coefficients ($beta1, beta2 and beta3)$.

In Model 2, a Type I space-time dependent structure was used where all parameters are similar to where they are in the "the space-time cube"^31^. In Model 3, a Type II space-time dependent structure was used where the temporal parameters in each neighbourhood *i*, are temporally smooth. The time trend pattern represented by the temporal parameters in area *j* ($i\neq j$) are not assumed to be dependent similar to each other, even in the case *i* and *j* are spatially contiguous^31^. In Model 4, a Type III space-time dependent structure was used where the area-specific parameters are spatially smooth at each time point *t*. However, the spatial pattern represented by the area-specific parameters at time point *t* and the spatial pattern represented by the area-specific parameters at time point g $(t\neq g$) is not assumed to be dependent on or similar to each other, even in the case where *t* and *g* are close^31^.

**WINBUGS Code** (Model 3: Space-time inseparable model with Type II interaction effect).

for (i in 1:N) {

for (t in 1:T) {

y[i,t] ~ dpois(mu[i,t]) # response variable Poisson likelikhood for the covid case count in #neighbourhoods i in time t

mu[i,t] <- pop[i]*theta[i,t] ## Population is assumed to be constant over time.

log(theta[i,t]) <- alpha + beta1*EDU[i,t] + beta2*LST[i,t] + beta3*IMMI[i,t] + S[i] + U[i] + v[t] + delta [i,t]

RR[i,t] <- exp(delta[i,t]) ## Spatiotemporal Relative Risk

} #close the for-loop over T

} #close the for-loop over N

for (i in 1:N) {

delta[i,1:T] ~ car.normal(tm.adj[],tm.weights[],tm.num[],prec.delta)

}

for (i in 1:N) {

U[i] ~ dnorm(0,prec.U)

}

S[1:N] ~ car.normal(sp.adj[], sp.weights[], sp.num[], prec.S)

v[1:T] ~ car.normal(tm.adj[],tm.weights[],tm.num[],prec.v)

# priors:

alpha ~ dflat()

beta1 ~ dnorm(0, 0.00001)

beta2 ~ dnorm(0, 0.00001)

beta3 ~ dnorm(0, 0.00001)

### set prior on standard deviation scale

sigma.S ~ dunif(0.0001,10) ## Standard Deviation

sigma.U ~ dunif(0.0001,10) ## Standard Deviation

sigma.v ~ dunif(0.0001,10) ## Standard Deviation

sigma.delta ~ dunif(0.0001,10) ## Standard Deviation

### convert standard deviations to precision as required by WinBUGS

prec.S <- pow(sigma.S,-2)

prec.U <- pow(sigma.U,-2)

prec.v <- pow(sigma.v,-2)

prec.delta <- pow(sigma.delta,-2)

sd.S <- sd(S[1:N]) # unconditional SD of the spatially structured random effects

var.S <- pow(sd.S,2) # unconditional variance of the spatially-structured random effects

sd.U <- sd(U[1:N]) # SD of the spatially-unstructured random effects

var.U <- pow(sd.U,2) # variance of the spatially-structured random effects variance partition coefficient/spatial fraction

sd.v <- sd(v[1:T])

var.v <- pow(sd.v,2) # variations of the temporal random effects

sd.delta <- sd(delta[1:N,1:T])

var.delta <- pow(sd.delta, 2)

vpc.SP <- (var.S + var.U)/(var.S + var.U + var.v + var.delta) ### VPC of spatial effects

vpc.TM <- var.v/(var.S + var.U + var.v + var.delta) ### VPC of temporal effects

vpc.SPTM <- var.delta/(var.S + var.U + var.v + var.delta) ### VPC of spatiotemporal effects

### the average COVID-19 rate per 1000 population per week in toronto

Toronto.average <- mean(theta[,])*1000

# Temporal Relative Risk

for (t in 1:T) {

temporal.RR[t] <- exp(v[t])

}

# Spatial Relative Risk

for (i in 1:N) {

spatial.RR[i] <- exp(S[i]+U[i])

}

}

**Fig. S2** Trace plot (top left), autocorrelation plot (top right), and Gelman-Rubin plot (bottom left) for checking of convergence, and density plot of the posterior distribution (bottom right) of the standard deviation of spatio-temporal interaction effects ($\theta_{it}$)


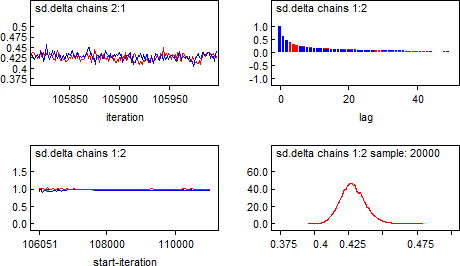


**Appendix 4**

**Sensitivity Analysis Results**

**Table S3**: Comparison of the DIC outputs from the sensitivity test.

|  | Dbar | Dhat | pD | DIC |
| --- | --- | --- | --- | --- |
| Model 3 with  Uniform Prior (0.01,10) | 52247.4 | 49015.6 | 3231.73 | 55479.1 |
| Sensitivity Test | | | | |
| Model with Gamma Prior (0.005,0.005) | 52263 | 49014.2 | 3249.63 | 55513 |
| Model with Uniform Prior (0.01,1000) | 52265.6 | 49013.2 | 3252.41 | 55518 |

**Table S4**: Comparison of the parameter estimates from the sensitivity test.

| Parameters | Model 3 with  Uniform Prior  (0.01,10) | Model with Gamma Prior (0.005,0.005) | Model with Uniform Prior (0.01,1000) |
| --- | --- | --- | --- |
| alpha | -8.556 | -8.557 | -8.556 |
| beta1 | -0.3258 | -0.3283 | -0.3282 |
| beta2 | 0.01516 | 0.0148 | 0.01552 |
| beta3 | 0.08472 | 0.08279 | 0.08244 |
| var.S | 0.04108 | 0.05567 | 0.0573 |
| var.U | 0.05079 | 0.0368 | 0.03744 |
| var.delta | 0.1834 | 0.1833 | 0.1838 |
| var.v | 3.481 | 3.454 | 3.48 |
| sd.S | 0.2001 | 0.2345 | 0.2381 |
| sd.U | 0.2237 | 0.1891 | 0.1909 |
| sd.delta | 0.4282 | 0.428 | 0.4286 |
| sd.v | 1.865 | 1.858 | 1.865 |

**Appendix 5: Map validation**

**Fig. S3: Comparison of the resulting Spatiotemporal trends and risk maps with the 4-week post cumulative new cases. (a) map of spatiotemporal trend, (b) spatial risk derived from the Bayesian models, (c) cumulative number of new cases during the post-study period.**

**
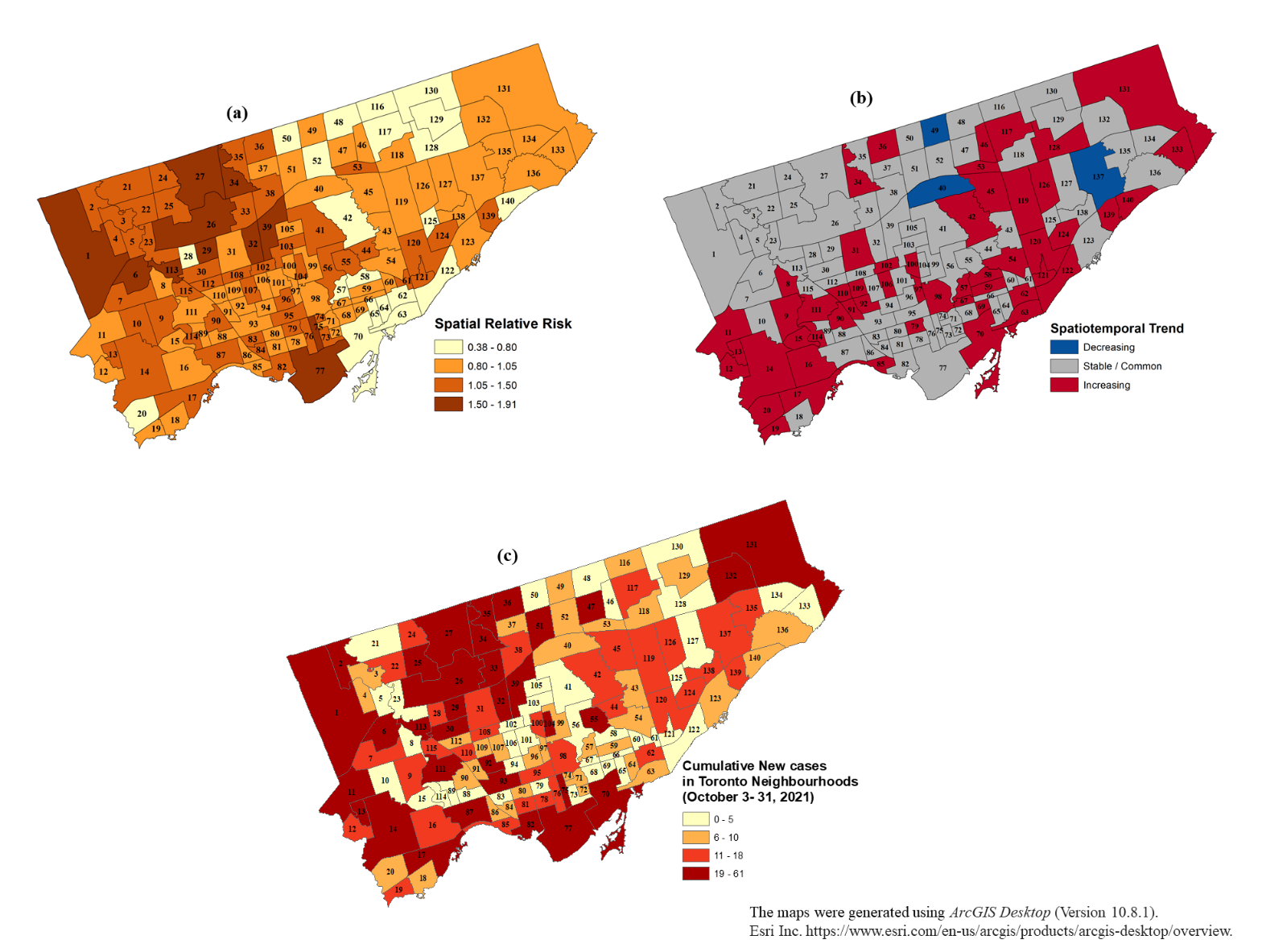
**

**References**

1. USGS- Landsat. Published 2021. https://www.usgs.gov/core-science-systems/nli/landsat

2. What are the acquisition schedules for the Landsat satellites? | U.S. Geological Survey. Accessed January 5, 2022. https://www.usgs.gov/faqs/what-are-acquisition-schedules-landsat-satellites

3. Land surface temperature and emissivity estimation from passive sensor data: Theory and practice-current trends: International Journal of Remote Sensing: Vol 23, No 13. Accessed December 1, 2021. https://www-tandfonline-com.myaccess.library.utoronto.ca/doi/abs/10.1080/01431160110115041?casa_token=cXxRH4DgOy4AAAAA:bbC37qfXy1SsUdnSa6fAS-LcIOZ0jtryoOCiDjsJ0oAKdquA7gAwJoZNkZO5yMNekZs_KHpQtglU9A

4. Jiménez-Muñoz JC, Sobrino JA, Skoković D, Mattar C, Cristóbal J. Land Surface Temperature Retrieval Methods From Landsat-8 Thermal Infrared Sensor Data. *IEEE Geoscience and Remote Sensing Letters*. 2014;11(10):1840-1843. doi:10.1109/LGRS.2014.2312032

5. Danodia A, Nikam B, Kumar S, R. P. *LAND SURFACE TEMPERATURE RETRIEVAL BY RADIATIVE TRANSFER EQUATION AND SINGLE CHANNEL ALGORITHMS USING LANDSAT-8 SATELLITE DATA*.; 2017.

6. Weng Q, Fu P, Gao F. Generating daily land surface temperature at Landsat resolution by fusing Landsat and MODIS data. *Remote Sensing of Environment*. 2014;145:55-67. doi:10.1016/j.rse.2014.02.003

7. Coll C, Caselles V. A split-window algorithm for land surface temperature from advanced very high resolution radiometer data: Validation and algorithm comparison. *Journal of Geophysical Research: Atmospheres*. 1997;102(D14):16697-16713. doi:10.1029/97JD00929

8. Sobrino JA, Jimenez-Munoz JC, Soria G, et al. Land Surface Emissivity Retrieval From Different VNIR and TIR Sensors. *IEEE Transactions on Geoscience and Remote Sensing*. 2008;46(2):316-327. doi:10.1109/TGRS.2007.904834

9. Sekertekin A, Bonafoni S. Land Surface Temperature Retrieval from Landsat 5, 7, and 8 over Rural Areas: Assessment of Different Retrieval Algorithms and Emissivity Models and Toolbox Implementation. *Remote Sensing*. 2020;12(2):294. doi:10.3390/rs12020294

10. Neinavaz E, Skidmore AK, Darvishzadeh R. Effects of prediction accuracy of the proportion of vegetation cover on land surface emissivity and temperature using the NDVI threshold method. *International Journal of Applied Earth Observation and Geoinformation*. 2020;85:101984. doi:10.1016/j.jag.2019.101984

11. Yin CL, Meng F, Yu QR. Calculation of land surface emissivity and retrieval of land surface temperature based on a spectral mixing model. *Infrared Physics & Technology*. 2020;108:103333. doi:10.1016/j.infrared.2020.103333

12. Sobrino JA, Jiménez-Muñoz JC, Paolini L. Land surface temperature retrieval from LANDSAT TM 5. *Remote Sensing of Environment*. Published online 2004. doi:10.1016/j.rse.2004.02.003

13. Atmospheric Correction Parameter Calculator. Accessed December 1, 2021. https://atmcorr.gsfc.nasa.gov/
